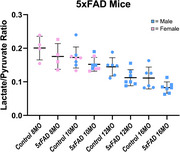# Detecting Metabolic Changes in Aging Progression and Alzheimer's Disease Using Non‐Invasive Imaging

**DOI:** 10.1002/alz70856_096944

**Published:** 2025-12-24

**Authors:** Julia R Zickus, José S Enriquez, Muxin Wang, Xudong Qiu, Sunil Goodwani, Morgan McReynolds, David Piwnica‐Worms, Seth T Gammon, Jim Ray, Pratip Bhattacharya

**Affiliations:** ^1^ University of Texas MD Anderson Cancer Center, Houston, TX, USA; ^2^ UT MD Anderson UT Health Houston GSBS, Houston, TX, USA; ^3^ The Neurodegeneration Consortium, UT MD Anderson Cancer Center, Houston, TX, USA

## Abstract

**Background:**

Recent metabolic research in Alzheimer's Disease has revealed glucose hypo metabolism as a potential biomarker in dementia pathology. By employing hyperpolarized (HP) pyruvate, the conventional MRI signal is enhanced by over 10,000 folds, enabling real‐time interrogation of the metabolic pathways. The development of biomarkers for early diagnosis of AD as well as imaging the progression of the disease is essential for effective therapeutic development and prevention at early stages of dementia. If successful, the HP MRI‐based metabolic imaging can be readily translated to the clinic and significantly impact AD patients.

**Method:**

HP‐MR was employed to measure the real‐time metabolic conversion of injected Dynamic Nuclear Polarization hyperpolarized 1‐^13^ C pyruvate to lactate in the brain *in vivo*. This *in vivo* imaging was performed on a 7T MRI animal scanner using multiple AD mouse models at multiple time points throughout their lifespan. After imaging, these mice were sacrificed, and brain tissues were excised for *ex vivo* metabolomics by nuclear magnetic resonance (NMR) spectroscopy.

**Result:**

The HP lactate‐to‐pyruvate ratio is decreased in the AD model mice, indicating reduced glycolytic activity. NMR metabolomics data of the brain indicates significant differences in the concentrations of choline, creatine, malate, glutamate, and N‐acetyl aspartate (NAA) all being decreased in 16‐month‐old male AD mice, with trending decreases in taurine and glutamine. Similarly, in 8‐month‐old Tau males, there were significant decreases in the concentrations of taurine, malate, glutamate, and NAA in the brain.

**Conclusion:**

The lactate‐to‐pyruvate ratio is decreased in the AD model mice, indicating altered glycolytic activity. This decrease is also observed as the mice age. This ratio decreases from 10 to 16 months in the males, and 8 to 10 months in the females, in both the control and 5xFAD mice. This provides an interesting aging component to this study. The preliminary metabolomics data seems promising to detect affected brain metabolites, but more samples at multiple ages are currently being processed including younger cohorts of the AD model mice. Future work will involve immunohistopathological validation along with optical imaging of the retina in these animal cohorts as well as repeating the HP‐MR experiments under therapeutic perturbation.